# Tumour-amplified kinase *BTAK* is amplified and overexpressed in gastric cancers with possible involvement in aneuploid formation

**DOI:** 10.1054/bjoc.2000.1684

**Published:** 2001-03

**Authors:** C Sakakura, A Hagiwara, R Yasuoka, Y Fujita, M Nakanishi, K Masuda, K Shimomura, Y Nakamura, J Inazawa, T Abe, H Yamagishi

**Affiliations:** Department of Digestive Surgery; Department of Hygiene, Kyoto Prefectural University of Medicine, Kamigyo-ku, Kyoto, Kawaramachi-dori, 602-8566; Human Genome Center, Institute of Medical Science, Tokyo University, Minato-ku, Tokyo, 108-8639, Japan

**Keywords:** ploidy pattern, amplification, overexpression, *BTAK*, gastric cancer

## Abstract

Our recent analysis of gastric cancers using comparative genomic hybridization (CGH) revealed a novel high frequent copy number increase in the long arm of chromosome 20. Tumour-amplified kinase *BTAK* was recently cloned from breast cancers and mapped on 20q13 as a target gene for this amplification in human breast cancers. In the study presented here, we analysed *BTAK* copy-number and expression, and their relation to the ploidy pattern in 72 primary gastric cancers. Furthermore, wild-type *BTAK* and its deletion mutants were transfected to gastric cancers to examine changes in cell proliferation and DNA ploidy pattern. Evaluation of 72 unselected primary gastric cancers found *BTAK* amplification in 5% and overexpression in more than 50%. All four clinical samples with *BTAK* amplification showed aneuploidy and poor prognosis. Transfection of *BTAK* in near-diploid gastric cancers induced another aneuploid cell population. In contrast, the c-terminal-deleted mutant of *BTAK* induced no effect in DNA ploidy pattern and inhibited gastric cancer cell proliferation. These results suggest that *BTAK* may be involved in gastric cancer cell aneuploid formation, and is a candidate gene for the increase in the number of copies of the 20q, and thus may contribute to an increase in the malignant phenotype of gastric cancer. © 2001 Cancer Research Campaign http://www.bjcancer.com


British Journal of Cancer (2001) **84**(6), 824–831
© 2001 Cancer Research Campaign
doi: 10.1054/ bjoc.2000.1684, available online at http://www.idealibrary.com on IDEAL

http://www.bjcancer.com

### Tumour-amplified kinase *BTAK* is amplified and overexpressed in gastric cancers with possible involvement in aneuploid formation

**C Sakakura[1,2], A Hagiwara[1], R Yasuoka[1,2], Y Fujita[1,2], M Nakanishi[1,2], K Masuda[2], K Shimomura[1], Y Nakamura[3], J Inazawa[2,3], T Abe[2] and H Yamagishi[1]**

[1] Department of Digestive Surgery; [2] Department of Hygiene, Kyoto Prefectural University of Medicine, Kamigyo-ku, Kawaramachi-dori, Kyoto 602-8566; [3] Human Genome Center, Institute of Medical Science, Tokyo University, Minato-ku, Tokyo, 108-8639, Japan

**Summary** Our recent analysis of gastric cancers using comparative genomic hybridization (CGH) revealed a novel high frequent copy number increase in the long arm of chromosome 20. Tumour-amplified kinase *BTAK* was recently cloned from breast cancers and mapped on 20q13 as a target gene for this amplification in human breast cancers. In the study presented here, we analysed *BTAK* copy-number and expression, and their relation to the ploidy pattern in 72 primary gastric cancers. Furthermore, wild-type *BTAK* and its deletion mutants were transfected to gastric cancers to examine changes in cell proliferation and DNA ploidy pattern. Evaluation of 72 unselected primary gastric cancers found *BTAK* amplification in 5% and overexpression in more than 50%. All four clinical samples with *BTAK* amplification showed aneuploidy and poor prognosis. Transfection of *BTAK* in near-diploid gastric cancers induced another aneuploid cell population. In contrast, the c-terminal-deleted mutant of *BTAK* induced no effect in DNA ploidy pattern and inhibited gastric cancer cell proliferation. These results suggest that *BTAK* may be involved in gastric cancer cell aneuploid formation, and is a candidate gene for the increase in the number of copies of the 20q, and thus may contribute to an increase in the malignant phenotype of gastric cancer. © 2001 Cancer Research Campaign http://www. bjcancer.com

**Keywords**: ploidy pattern; amplification; overexpression; *BTAK*; gastric cancer

Gastric cancer is the second most common cause of cancer-related death in the world (Whelan et al, 1993), but the genetic changes underlying the development and progression of this type of carcinoma are poorly understood. We previously analysed chromosomal aberrations in 72 primary gastric cancers by means of comparative genomic hybridization (CGH) and identified several high-frequency regions, including 20q with an increased DNA copy-number whose target genes are as yet unknown (Sakakura et al, 1999). Increased DNA copy-number of 20q has also been observed in bladder, colon and breast cancers, while amplification on 20q12–13 correlates with the malignant phenotype of breast cancer (Kallioniemi et al, 1994; Tanner et al, 1995; Vooter et al, 1995; Ried et al, 1996). Recently, several candidate genes for this amplicon, *AIB1*, *BTAK* and *Dcr3* have been isolated (Anzick et al, 1997; Sen et al, 1997; Pitti et al, 1998; Zhou et al, 1998).

*BTAK* (Breast tumour-amplified kinase, identical to aurora2, ARK1, AIK, STK15 in alternative names) was cloned from breast cancers and mapped on 20q13 as a target gene for this amplification in human breast cancers and for analysis of its function (Kimura et al, 1997; Sen et al, 1997; Bischoff et al, 1998; Shindo et al, 1998; Zhou et al, 1998). The findings of these studies that *BTAK* is critical in leading to centrosome amplification, chromosome instability, chromosome segregation process and transformation in mammalian cells. Its role in gastric cancer progression, however, has not yet been clarified.

*Received 9 June 2000*
*Revised 31 October 2000*
*Accepted 30 November 2000*

*Correspondence to:* C Sakakura

Previous studies have suggested that most cancers with aneuploidy show a more malignant phenotype than those with diploid (Estiban et al, 1999; Ikeguchi et al, 1999), but the causes of aneuploidy have not yet been clarified. Identification of mutations in the mitotic checkpoint gene *BUB1* in human cancers was the first evidence that acquired aneuploidy may be a specific dividing force in tumour progression, rather than an epiphenomenon of this disease (Cahill et al, 1998), but its mechanism remains unclear. Another candidate gene is *BTAK*.

To determine the involvement of *BTAK* in gastric cancer aneuploidy formation, we analysed the amplification and overexpression of *BTAK* and its relation to the DNA ploidy pattern in gastric cancer. Furthermore, transfection of wild-type *BTAK* or its deletion mutants was used to analyse the role of *BTAK* in anuploid formation in gastric cancer cells.

### MATERIALS AND METHODS

### Tumour samples and cell lines

All 72 primary gastric cancers were surgically resected specimens. Their distribution in clinical stage was as follows: stage I, 14 cases; stage II, 17 cases; stage III, 32 cases; stage IV, 9 cases. High-molecular-weight DNA was isolated from homogenized tumour specimens using standard protocols. DNA was isolated from the peripheral blood of a normal male donor as a reference standard for CGH. For Southern blot analysis, high-molecular-weight DNA also was isolated from six gastric cancer cell lines, MKN1, MKN28, MKN45, MKN74, KATO-III and NUGC-3. The origin, nature and the number of chromosomes are as follows: MKN1 – metastatic lymph node, adenosquamous carcinoma, 39; MKN28 – metastatic lymph node, moderately differentiated adenocarcinoma, 59–80; MKN45 – metastatic lymph node, poorly differentiated adenocarcinoma, 44; MKN74 – metastatic tumour, moderately differentiated adenocarcinoma, 36; KATO-III – pleural effusion, signet ring cell carcinoma, 88; and NUGC-3 – metastatic tumour, poorly differentiated adenocarcinoma, 58. These were maintained in RPMI-1640 containing 10% foetal calf serum (FCS). These cell lines were purchased from Riken cell bank in Japan.

### Southern blot analysis

Genomic Southern blot was performed as we described previously (Sakakura et al, 1999). In brief, *Eco*RI-digested tumour DNA and human placental DNA (5 µg) were electrophoresed in a 0.8% agarose gel and transferred to a nylon membrane (Hybond-N, Amersham Pharmacia Biotech UK Ltd, Buckinghamshire). A 50 ng aliquot of each probe was labeled with $\alpha^{32}$P-dCTP using random primers, then hybridized to the prehybridized filter. We analysed signals with a BAS 2000 image analyser (Fuji, Tokyo) and calculated the degree of amplification. A probe for β-actin was used to control for loading error. BTAK probe is 248bp cDNA fragment from nt 1751–nt1998. This cDNA probe was used for both Southern and Northern blot analysis.

### Northern blot analysis

Northern blot was performed as we described previously (Sakakura et al, 1996). In brief, total cellular RNA was prepared by the guanidine isothiocyanate-phenol-chloroform procedure. Selection of poly $(A)^+$ RNA was performed by an oligo dT column, then fractionated on 1% agarose/2.2 M formaldehyde gels. Probes were labeled with $^{32}$P by random priming. Each blot was hybridized with probes for *BTAK* and β-actin, as described previously (Sakakura et al, 1996, 1999, 2000). We analysed signals with a BAS 2000 image analyser and calculated the degree of overexpression compared to control.

### Fluorescence in situ hybridization (FISH)

FISH was carried out as described previously (Ishino et al, 1998). Interphase nuclei were fixed in methanol and acetic acid (3:1) and dropped onto microscope slides. 1 µl of Cot-1 was added to 9 µl of probe hybridization solution. The final mixture was denatured at 75˚C for 10 min, cooled on ice for 5 min, then mixed with an equal volume of 4 × SSC containing 20% dextran sulfate. The hybridization mixture was placed on denatured slides, covered with Parafilm, and incubated in a humidified box for 16 –24 h. After being washed in 50% formamide/2 × SSC, 2 × SSC, and 1 × SSC. Slides were counterstained with DAPI (1 µg $ml^{-1}$) and mounted in an antifade solution containing p-phenylenediamine (PPD). Fluorescence images were captured with a Zeiss axiophot microscope equipped with a charge-coupled device camera. A centromeric probe on chromosome 20, D20Z1 (Oncor, Gaitherburg) was used as an internal control. The BTAK FISH probe was obtained by screening BAC clones with a BTAK cDNA probe.

### Semi-quantitative reverse transcriptase-polymerase chain reaction (RT-PCR)

Semi-quantitative RT-PCR was performed following the procedure of Nakayama et al (1992). cDNA was produced from total RNA by using a Superscript preamplification system (BRL, Bethesda) and following the procedures suggested by the manufacturer. RNA was heated to 70˚C for 10 min in 14 µl of duethylpyrocarbonate-treated water containing 0.5 µg oligo (dT). Synthesis buffer (10 ×), 2 µl 10 mM dNTP mix, 2 µl 0.1 M DTT, and reverse transcriptase (Superscript RT, 200 U $\mu l^{-1}$) were added to the sample. The resulting reaction mixture was incubated at 42˚C for 50 min, and reaction was terminated by incubating the mixture at 90˚C for 5 min. The 100 µl reaction mixture containing 2 µl first-strand cDNA was used for PCR. PCR was performed for 10 –35 cycles in a Perkin-Elmer (Foster City, California) thermal cycler using a step cycle program at 94˚C for 30 s, 55˚C for 30 s, and 72˚C for 30 s. After PCR, 10 µl of the reaction mixture was electrophoresed on a 1.5% agarose gel. The ethidium bromide fluorescence intensity of the bands was measured with the NIH image program. A ratio of *BTAK* overexpression was calculated by comparing the signal intensities of β-actin and of BTAK in normal mucosa and cancerous tissue. Primer sequences used for RT-PCR are as follows: BTAK: forward primer, 5′-TAGGCATGGTGTCTTCAC-3′, reverse primer, 5′ -CATTCCTAAAGGAATGCC-3′; β-actin: forward primer, 5′-GAGCTGCGTGTGGCTCCCGAGG-3′, reverse primer, 5′-CGCAGGATGGCATGGGGGAGGGCATACCCC-3′.

### Western blot analysis

Western blot analysis was performed as described previously (Sakakura et al, 1996). Cells were washed twice with PBS and lysed in 1% Triton X-100, 0.15M NaCl and 10 mM Tris HCl, pH 7.4, with 50 µg $ml^{-1}$ PMSF at 4˚C for 60 min. Lysates were centrifuged at 10 000 rpm for 10 min. Samples were boiled in SDS sample buffer for 5 min before running on a 10–20% SDS-polyacrylamide gel after overnight transfer of SDS-polyacrylamide gel to nitrocellulose membrane. After blocking the blot in 3% BAS for 2 h at room temperature, immunodetection of BTAK was performed with monoclonal antibody IAK1 (mouse monoclonal antibody, Transduction Laboratories, Lexington) at 1:250 dilution for 24 h at room temperature. Horseradish peroxidase-conjugated anti-mouse IgG antibody was incubated with the blot at 1:1000 dilution in PBS with 1% non-fat dry milk. The blot was visualized with ECL kit (Amersham).

### Vector constructs

The expression vector pCX2neo BTAK was used for all experiments. This vector places the human BTAK under the transcriptional control of β-actin promoter and CMV enhancer as described previously (Niwa et al, 1991; Sakakura et al, 1994). Several deletion mutants were prepared as shown in Figure 5A.

### Transfection

Gastric cancer cell line MKN1, MKN45 and MKN74 cells were stably transfected with pCX2neoBTAK or native pCX2neo vector using Lipofectin (GIBCO, Grand Island) as described previously (Sakakura et al, 1994, 1996). These gastric cancer cell lines MKN1, MKN45 and MKN74 are near-diploid, and we have selected the near-diploid cells for the experiments of BTAK transfection. Cells ($2 \times 10^4$) were plated on 35-mm dishes in serum-free media and transfected with 2 µg of pCX2neoBTAK or pCX2neo

© 2001 Cancer Research Campaign

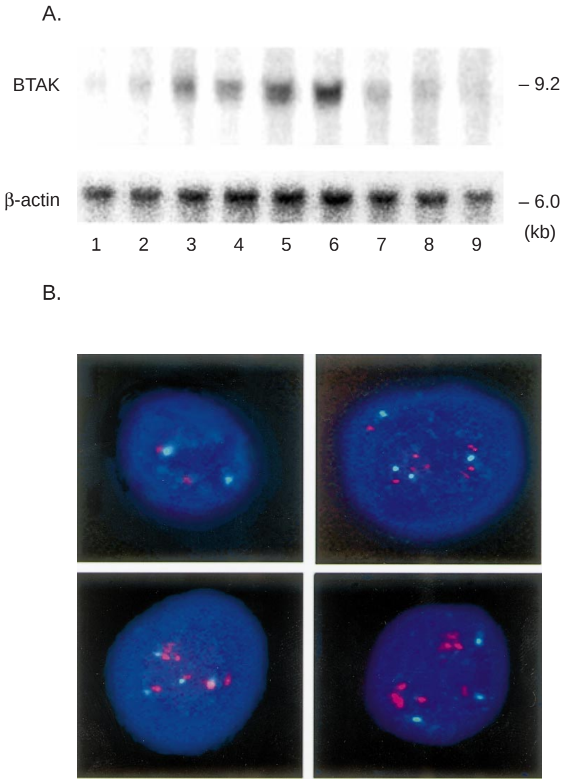

**Figure 1** (**A**) Southern blot analysis demonstrates amplification of *BTAK* in primary gastric cancers. 5 μg of *Eco* R-I-digested DNA was loaded per lane. The degree of amplification was calculated with a BAS4000 image analyser. Rehybridization was performed on the same membrane with β-actin (*ACTB*) as a control for DNA loading error. (**B**) Bicolour FISH analysis demonstrates *BTAK* amplification (red signals) in gastric cancer cells. Numerous copies of *BTAK* were resolved in interphase nuclei. The digoxigenin-labelled *BTAK* P1 probe was hybridized with a biotinylated reference centromeric probe on chromosome 20 which appears green. Representative nuclei of gastric cancers show various patterns of signal exhibiting: (**a**) disomy; (**b, c** and **d**) high-level amplification.

DNA. Each transfection was performed two separate times to ensure the generation of unique clones. At 24 h following transfection, RPMI with 20% FCS was added to the plates. After 48 h, the cells were trypsinized and transferred to 100-mm dishes, and the following day media was supplemented with 1 mg $ml^{-1}$ G418 (GIBCO) and cultured for 4 weeks. Individual G418-resistant clones were generated by limited dilution.

*BTAK* expression was determined by RT-PCR. For RT-PCR, primer sequence sense primer 5′-GCAACGTGCTGGTTGTTG-3′ corresponds to the sequence in the globin gene in pCX2neo vector. Antisense primer site is as shown in Figure 5A.

### Measurement of DNA ploidy pattern by flow cytometry

Preparation of the cell suspensions and measurement of nuclear fluorescence were performed according to the method described previously (Ohta et al, 1995). Briefly, the tumour material was mechanically minced with scissors and the tissue pieces were suspended in Hank's balanced solution, passed 10–20 times through a sharp-edged glass pipette, and the resultant cell suspension filtered through gauze. The cell pellet ($1 \times 10^6$ cells), obtained by 200 ***g*** centrifugation, was suspended in 1 ml of fluorochrome solution (50 mg $ml^{-1}$ propidium iodine, 0.1% sodium citrate, 0.1% Triton X-100). Samples were plated overnight in the dark at 4˚C,

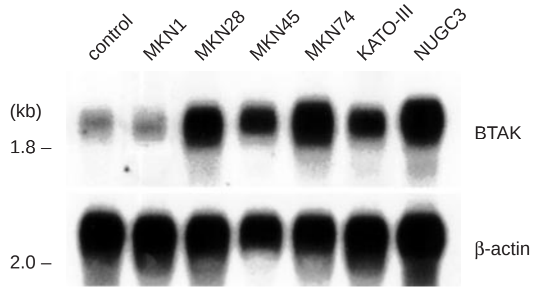

**Figure 2** Northern blot analysis of *BTAK* demonstrates increased expression relative to normal gastric mucosa (control) in the gastric cancer cell lincs MKN1, MKN 28, MKN 45, MKN 74, KATO-III and NUGC-3. The blot was hybridized sequentially with the indicated probes to compare *BTAK* expression with that of β-actin.

and fluorescence of individual nuclei was measured using an FACS flow cytometer (FACS Vantage™, Becton-Dikinson) equipped with an argon ion laser, with a propidium iodide extraction of 488 nm and fluorescence emission of 590 nm.

### Statistical methods for analysis

Statistical analysis was performed using version 4.0 of the NAP system programmed by Aoki (1989). The first objective of the statistical analysis was to examine the influence of *BTAK* amplification on DNA ploidy pattern with unpaired *t*-test. The various groups of patients were compared by means of either $\chi^2$ test or Mann-Whitney U test. Results with *P* values of less than 0.05 were considered statistically significant.

### RESULTS

### Southern blot analysis

Southern blot analysis demonstrated amplification in four cases (5%). Typical data are shown in Figure 1A. Intense bands were observed in lanes 3, 4, 5 and 6 (3.5-fold, 5.1-fold, 5.8-fold and 6.3-fold, respectively compared to control lane 1). A β-actin probe was used as a control for loading errors in the lanes.

### Fluorescence in situ hybridization

Tumours were categorized into three groups according to *BTAK* copy-number, using the classification of Meltzer et al low – fewer than four copies or no increase in number; moderate – 4 – 6 copies per cell or a 1.5–3.0-fold increased relative copy-number; high – more than six copies per cell or > 3-fold relative copy-number increase. Thirty-four of 72 gastric cancers showed an increase in the number of copies. Typical data is shown in Figure 1B. Numerous copies of *BTAK* (red signals) were resolved in large interphase nuclei (a = control, normal pattern of peripheral lymphocyte; b, c and d = high-level amplification). Green signal indicates internal control of chromosome 20. FISH analysis revealed high-level amplification in four cases (5%), moderate-level amplification in 11 cases (15%), and low-level amplification in 19 cases (28%).

*© 2001 Cancer Research Campaign*

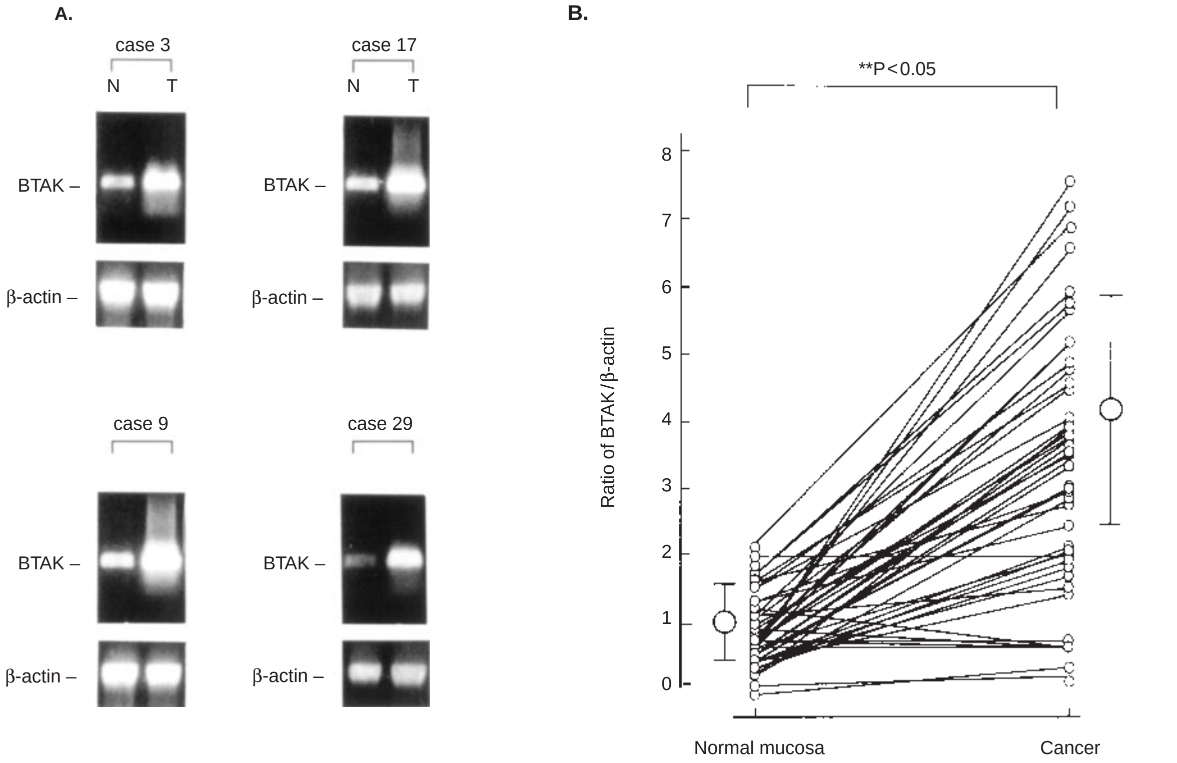

**Figure 3** (**A**) RT-PCR analysis of BTAK/β-actin mRNA expression in normal gastric mucosa and cancerous tissue. Four representative cases of BTAK/β-actin expression are shown. A (T = tumour; N = paired non-cancerous tissue). (B) BTAK/β-actin ratios in normal gastric mucosa and cancerous tissue. Figures represent the mean ± standard deviation* $P < 0.05$, two tailed *t*-test

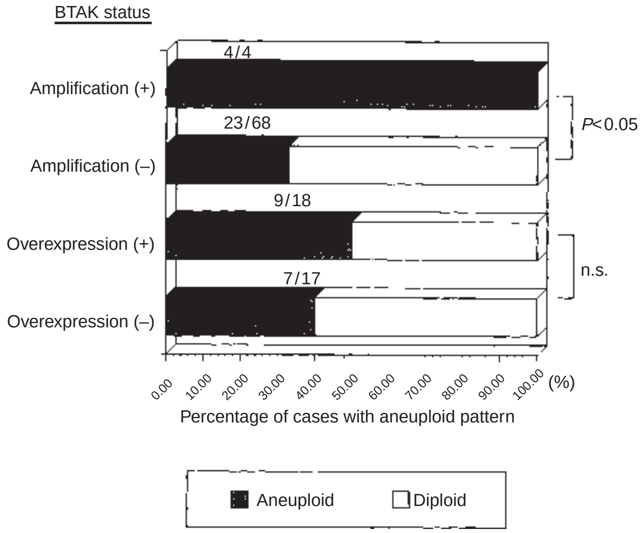

**Figure 4** (**A**) Relationship between DNA ploidy and *BTAK* amplification, $^*P < 0.05$, two tailed *t*-test. (B) Relationship between DNA ploidy and *BTAK* overexpression, $^*P < 0.07$, not significant

### Northern blot analysis

*BTAK* expression was examined in six gastric cancers and compared with β-actin expression by Northern blotting. These cells did not show high-level amplification (data not shown), but indirectly proportional to amplification, *BTAK* was highly overexpressed in all lines except MKN1 Figure 2.

### Semi-quantitative RT-PCR

Informative results were obtained in 35 of 72 cases. Representative cases of *BTAK*/β-actin expression are shown in Figure 3A. Signals observed in cancerous tissues were intensive in comparison with the signals of normal gastric mucosa in cases 3, 9, 17 and 29. *BTAK*/β-actin ratios are plotted in Figure 3B. *BTAK* expression in primary gastric cancers increased relative to that in normal gastric mucosa in 18 of 35 cases (51%, $P < 0.05$, two tailed *t*-test).

### Relationship between DNA ploidy pattern and BTAK amplification or its overexpression

All four cases with *BTAK* amplification showed aneuploidy formation. The incidence of cases of aneuploidy with *BTAK* amplification was significantly higher than that of cases without amplification ($P < 0.05$). The incidence of cases of aneuploidy with *BTAK* overexpression was somewhat higher than that of cases without *BTAK* overexpression, but not significantly (Figure 4).

### Schematic representation of BTAK-deletion mutants and expression of the transfected genes in stable transfect

Schematic representation of *BTAK* and its deletion mutants are shown in Figure 5A. Expression of the transfected genes in the cells was confirmed by RT-PCR analysis. The locations of the antisense primers used for the analysis are indicated in Figure 5A. Fragments of the expected size were amplified in all cases in each cell line: 1201 bp for wild-type BTAK, 913 for delBTAK1 (BTAKΔ 354 – 403), and 672 bp for delBTAK2 (BTAKΔ55–134)

© 2001 Cancer Research Campaign 

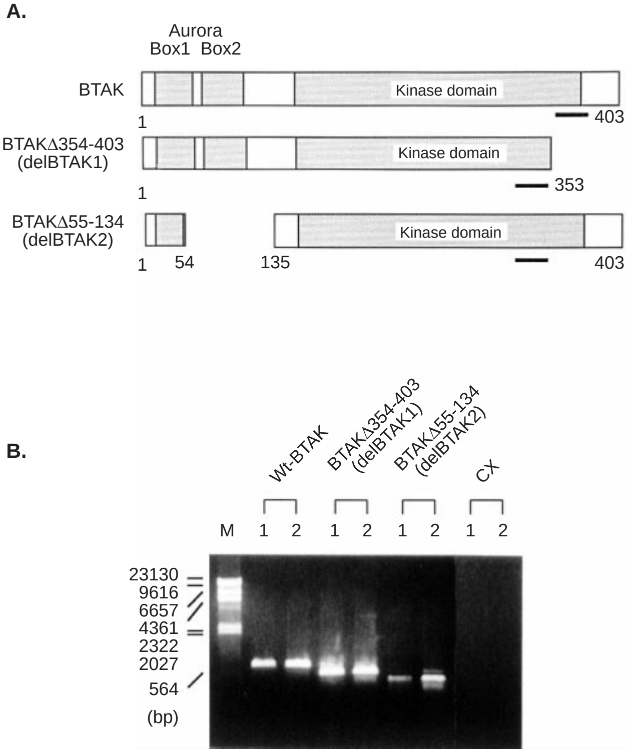

**Figure 5** (**A**) Schematic representation of *BTAK* and its deletion mutants. Wild-type BTAK, delBTAK1 (BTAKΔ354 – 403), and delBTAK2 (BTAKΔ55–134) are represented by solid bars. The figures indicate the number of amino acids in the polypeptides. The antisense primers used for RT-PCR analysis are indicated by bars. (**B**) RT-PCR analysis of transfected genes in each stable transfectant

(Figure 5B). Two independent clones of each stable transfectant were selected and their expression examined. None of the PCR-product was detected in cells transfected with the plasmid harbouring only the neomycin-resistant gene.

### Morphological changes in stable transfectant

Three independent experiments with cells stably transfected with wild-type BTAK showed that these cells have a large body, with a multiple nucleus in MKN1 cells (Figure 6A, 6B and 6C). Transfection and overexpression of *BTAK* were detected in stable transfectants of gastric cancer cell lines as shown in Figure 5. Figure 6D shows the cells transfected with the plasmid harbouring only the neomycin-resistant gene.

### Changes of cell proliferation in stable transfectants

As shown in Figure 7A, MKN1 cells with delBTAK2 (BTAKΔ55–134) did not show any appreciable change in their growth rates, while MKN1 cells with BTAK showed a slight increase in their growth rates. In contrast, expression of delBTAK1 (BTAKΔ354 – 403) significantly prolonged the doubling time from 35–100 h. In the another cell line, MKN74, the same tendency was observed in each stable transfectant, but not in MKN45 and NUGC3.

### Measurement of DNA ploidy pattern by flow cytometry

The DNA content of aneuploid cells resulted in an unequivocal hyperdiploid DNA peak which was distinguishable from the diploid DNA peak in MKN1/BTAK cells, as shown in Figure 7B. As shown in Figure 7B, aneuploid cells were observed in only 5 –10% of parental MKN1 and MKN1/CX cells (control), but in 30 – 40% of MKN1/BTAK. The percentage of aneuploid cells in MKN1/BTAK cells was significantly higher than that in the parent MKN1 cells and MKN1/CX cells (control). In the another cell line, MKN74, the same tendency was observed in each stable transfectant.

### Western blot analysis

We examined expression of BTAK protein in three independent clones of MKN1 cells stably transfected with wild-type BTAK (clone 1, clone 2 and clone 3) by Western blot analysis. As shown in Figure 8, stable transfectants of BTAK showed strong expression of BTAK protein at molecular size of 46 kD compared to control cells, to which only neomycin-resistant genes were introduced. Anti-β-actin antibody was used to exclude loading error.

### DISCUSSION

Gene amplification of *MET*, *MYC*, *HST1/INT2*, *ERBB2* and other genes has been reported in gastric cancer as a marker of poor prognosis (Ranzani et al, 1990; Tahara, 1995). Our recent analysis using comparative genomic hybridization of chromosomal aberrations in gastric cancers has revealed an unusual frequency of increases in the number of copies in the long arm of chromosome 20, indicating that this region contains a novel amplified gene involved in gastric cancer progression. *BTAK* (identical to aurora2, ARK1, AIK, STK15 in alternative names) has been cloned on 20q13 as a candidate target gene for this amplification in human breast cancers (Kimura et al, 1997; Sen et al, 1997; Bischoff et al, 1998; Shindo et al, 1998; Zhou et al, 1998).

This gene is a cell-cycle regulated serine-threonine kinase and is the first example to suggest a link between centrosome integrity and cellular transformation (Bischoff et al, 1998; Bischoff and Dlowman, 1999). Until recently, no compelling connection could be established between the proteins involved in this process of chromosome seggregation and cancer. However, amplification and overexpression of *BTAK* cause centrosome amplification, deregulated duplication, and aneuploid formation, thus suggesting that it is implicated in the centrosome segregation abnormalities and aneuploidy seen in many cancer cell types (Zhou et al, 1998).

We examined the number of *BTAK* copies, their expression and their relation to DNA ploidy patterns in 72 primary gastric cancers. In all cases a gain on chromosome 20q detected by CGH could be confirmed by FISH, but the number of *BTAK* copies increased more than expected after CGH and high-level amplification of *BTAK* was identified in four cases (5%), and overexpression in more than 50% of the primary gastric cancers tested. *BTAK* amplification coincided with this overexpression, but many cases showed *BTAK* overexpression without amplification, suggesting that the expression of *BTAK* is likely to be regulated not only by gene amplification but also by other mechanisms such as transcriptional activation in human gastric cancers. These findings suggest that *BTAK* is one of the putative target genes in this region of frequent increases in the number of copies in primary gastric cancers. All cases with *BTAK* amplification showed aneuploidy. Cases with *BTAK*-overexpression showed the same tendency but not significantly. These results indicate that *BTAK* may be at least partly involved in gastric cancer aneuploidy.

 © 2001 Cancer Research Campaign

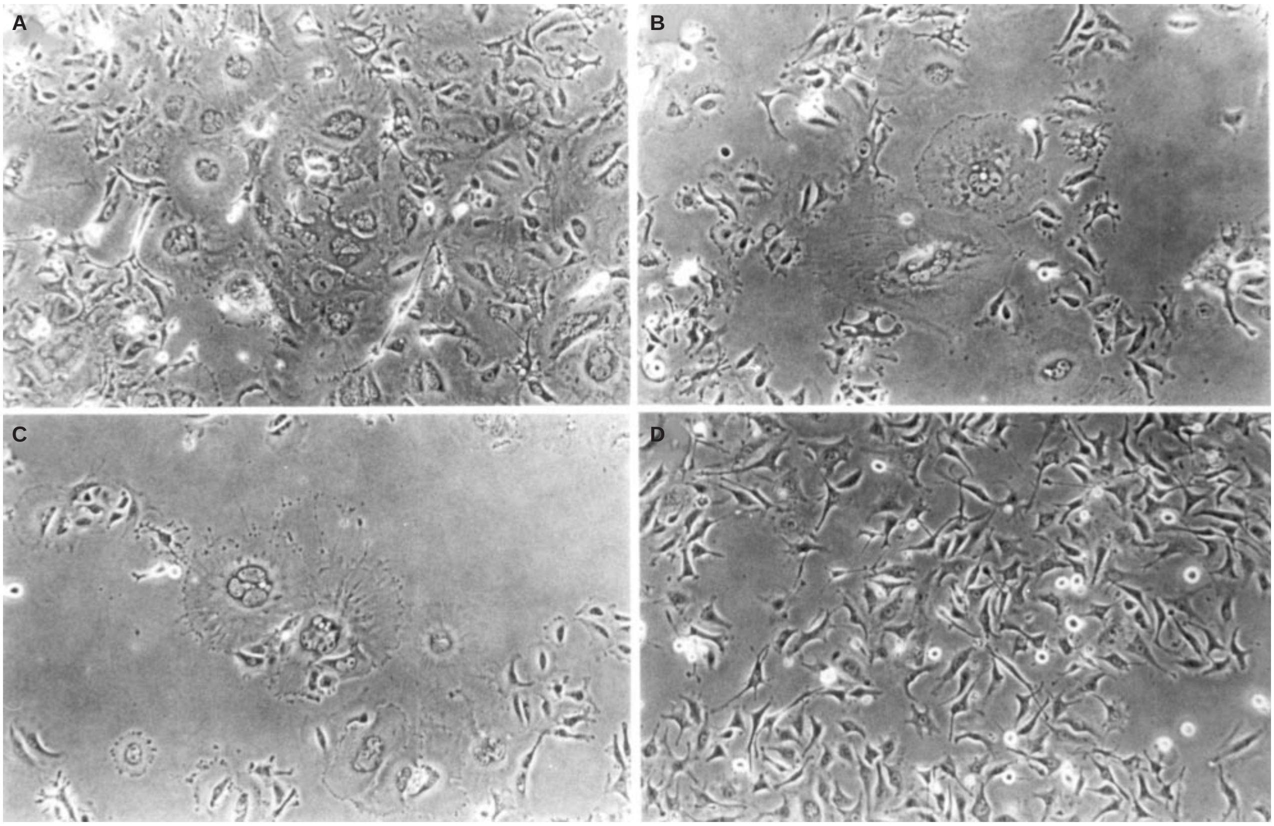

**Figure 6** Morphological changes in wild-type BTAK-transfected cells. (**A, B** and **C**) Stable transfectants of BTAK, (**D**) control

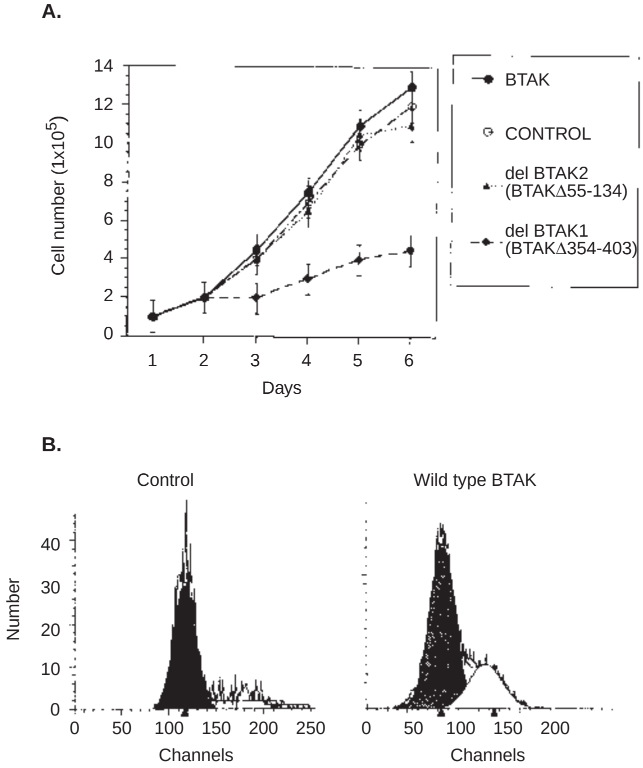

Next, we analysed the roles of *BTAK* using transfection of several kinds of deletion mutants in gastric cancer cell lines. After transfection was established, we compared morphological changes, DNA ploidy pattern and proliferation rate. Stable transfectants of wild-type *BTAK* showed large cells with large multiple nuclei, and overexpression of *BTAK* enhanced cell proliferation to some extent. In contrast, c-terminal deletion mutants inhibited cell proliferation, probably due to dominant negative effect. FACS analysis revealed the existence of another cell population, indicating that the ancuploid cell population appeared in a stable transfectant.

These findings as well as the observation of growth inhibition by deletion mutants and morphological changes in wild-type *BTAK*-stable transfectants, suggest that *BTAK* is, at least in part, involved in gastric cancer cell proliferation through chromosome segregation and aneuploidy formation. *BTAK* amplification and overexpression was frequently observed in aneuploid gastric cancer cells, but we hypothesize that *BTAK* is not the only cause of aneuploidy, since many genes could be involved in gastric cancer aneuploidy formation. For example, mutations found in the mitotic

**Figure 7** Cell proliferation and DNA ploidy pattern of BTAK-transfected cell. (A) Growth curve of gastric cancers MKN1. Cells were seeded in growth medium on day 0 and incubated for 72 h. Each stable transfectant of wild-type BTAK (●), del BTAK1 (▲), del BTAK2 (◆) and control (○) in fresh medium. The results represent the mean ± standard deviation from triplicate cultures. Student's *t*-test shows that difference between the stable transfectant of wild-type BTAK and that of del BTAK 2 to be statistically significant ($P < 0.05$). (**B**) FACS analysis. The DNA content of aneuploid cells resulted in an unequivocal hyperdiploid DNA peak which was distinguishable from the diploid DNA peak in MKN1/BTAK cells

*© 2001 Cancer Research Campaign* 

**Table 1** Clinicopathologic features of four cases with *BTAK* amplification

| | Age (years) gender | Clinical stage* | Borrman classification | Histological type | Recurrence survival |
|---|---|---|---|---|---|
| Case 1 | 43 M | P0H0N3T3 Stage IVa | 3 | Intestinal | Liver metastases 13 month |
| Case 2 | 63 F | P0H0N3T3 Stage IVa | 3 | Diffuse 9 month | Peritoneal dissemination |
| Case 3 | 58M | POH0N2T2 Stage IIIa | 3 | Diffuse | Liver metastases 11 month |
| Case 4 | 59 F | P0H0N3T2 Stage IIIb | 3 | Diffuse | Liver metastases 12 month |

* According to Japanese Gastric Cancer Classification

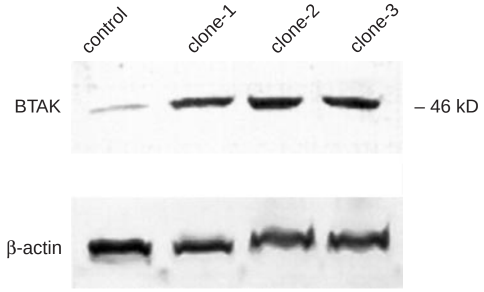

**Figure 8** BTAK expression in protein level in each wild-type BTAK stable transfectant. Fifty micrograms protein were used in each lane checkpoint gene *BUB1* in human cancers indicate that acquired aneuploidy may be a specific phenomenon in tumour progression, rather than an epiphenomenon of this disease (Cahill et al, 1998). *MET* is also known to mediate non-random chromosome duplication (Zhuang et al, 1998). These cancers have chromosome instability and in case of additional genetic changes easily become aneuploid even if the cancer cells are still diploid. This could in turn contribute to the rapid growth kinetics in the subset of gastric cancers.

Previous studies indicate that overexpression of *BTAK* is transforming only in the presence of co-amplification or mutation of additional genes (Zhou et al, 1998). Likewise in our study, *BTAK*-transfection induced aneuploid cells in MKN1 and MKN74, but not in MKN28 or NUGC3 cells. The morphologically changed cells would need to maintain an appropriate BTAK expression level or additional changes of other genes. Further studies will be needed to clarify the mechanisms of aneuploid formation.

A previous interphase FISH study demonstrated that amplification of chromosome 20q in breast cancer was complex and involved several distinct variably co-amplified segments derived from 20q11, 20q12, and 20q13 (Tirkkonen et al, 1998). In breast cancer, most instances of *BTAK* amplification were observed in conjunction with an increase in the number of 20q12 copies. Similarly, a complex amplification on 20q may occur in gastric cancers. Recently, the *AIB1* gene was localized on 20q12 as a putative target gene (Anzick et al, 1997; Sakakura et al., 2000), which may be involved in gastric cancer progression.

Recently we analysed amplification of putative target genes on 20q12–13 including *BTAK* (20q13) and *AIB1* (20q12). Coamplification of *BTAK* and *AIB1* was observed in four cases of Table 1 (data not shown), and these cases had poor prognosis than cases without BTAK amplification. We have not yet been able to clarify which gene is important in this region, but it appears that multiple genes on this locus are involved in the progression of gastric cancers. Further studies are now underway to clarify their respective roles.

In conclusion, dysregulation through *BTAK* gene amplification and overexpression provides a selective advantage for tumour growth and aneuploid formation in gastric cancers. A previous study indicated that most cancers with aneuploidy show a more malignant phenotype than diploid cancers (Estiban et al., 1999; Ikeguchi et al, 1999). As the incidence of *BTAK* amplification and overexpression in gastric cancers was found to be as high as in breast cancers and colon cancers, the frequent amplification and overexpression of *BTAK* may indicate that aneuploid formation confers a growth advantage even on gastric cancer cells. These observations suggest that the altered expression of *BTAK* may contribute to a more malignant phenotype of gastric cancer. Amplification and overexpression of *BTAK* appears to be important in chromosomal segregation and aneuploid formation, and may be involved in the development of a clinically aggressive subset of gastric cancer. In other words, *BTAK* may be a marker of poor prognosis.

### ACKNOWLEDGEMENTS

This work was supported by a Grant-in-Aid for Cancer Research from the Ministry of Health and Welfare and from the Ministry of Education, Science and Culture, and by grants from the Uehara Memorial Foundation and Sagawa Memorial Foundation, Japan.

### REFERENCES

Anzick SL, Kononen J, Walker R, Azorsa DO, Tanner MM, Guan XY, Sauter G, Kallioniemi OP, Trent JM and Meltzer PS (1997) AIB1, a steroid receptor coactivator amplified in breast and ovarian cancer. *Science* **277**: 965–968

Aoki S (1989) Reference Manual for Medical Statistical Analysis, 1st edn. (in Japanese) Igakushoin, Tokyo

Bischoff JR and Plowman GD (1999) The Aurora/Ip11p kinase family: regulators of chromosome segregation and cytokinesis. *Trends Cell Biol* **9**: 454 – 459

Bischoff JR, Anderson L, Zhu Y, Mossie K, Ng L, Souza B, Schryver B, Flangan P, Clairvoyuant F, Ginther C, Chan CS, Novotny M, Slamon DJ and Plowman GD (1998) A homologue of Drosophila aurora kinase is oncogenic and amplified in human colorectal cancers. *EMBO J* **17**: 3052–3065

Cahill DP, Lengauer C, Yu J, Riggins GJ, Willson JK, Markowitz SD, Kinzler KW and Vogelstein B (1998) Mutations of mitotic checkpoint genes in human cancers. *Nature* **392**: 300–303

 © 2001 Cancer Research Campaign

Estiban F, de Vega DS, Garcia R, Rodriguez R, Manzanares J, Almeida A and Tamames S (1999) DNA content by flow cytometry in gastric carcinoma: pathology, ploidy and prognosis. *Hepatogastroenterology* **46**: 2039–2043

Guan XY, Xu J, Anzick SL, Zhang H, Trent JM and Meltzer PS (1996) Hybrid selection of transcribed sequences from microdissected DNA: isolation of genes within amplified region at 20 q11– q13.2 in breast cancer. *Cancer Res* **56**: 3446–3450

Ikeguchi M, Oka S, Saito H, Kondo A, Tsujitani S, Maeta M and Kaibara N (1999a) Computerized nuclear morphometry: a new morphologic assessment for advanced gastric adenocarcinoma. *Ann Surg* **229**: 55–61

Ikeguchi M, Cai J, Yamane N, Maeta M and Kaibara N (1999b) Clinical significance of spontaneous apoptosis in advanced gastric adenocarcinoma. *Cancer* **85**: 2329–2335

Ishino S, Hashimoto N, Fushiki S, Date K, Mori T, Fujimoto M, Nakagawa Y, Ueda S, Abe T and Inazawa J (1998) Loss of material from chromosome arm 1p during malignant progression of meningioma revealed by fluorescent in situ hybridization. *Cancer* **83**: 360–366

Kallioniemi A, Kallioniemi OP, Piper J, Tanner M, Stokke T, Chen L, Smith HS, Pinkel D, Gray JW and Waldman FM (1994) Detection and mapping of amplified DNA sequences in breast cancer by comparative genomic hybridization. *Proc Natl Acad Sci USA* **91**: 2156–2160

Kimura M, Kotani S, Hattori T, Sumi N, Yoshioka T, Todokoro K and Okano Y (1997) Cell cycle-dependent expression and spindle pole localization of a novel human protein kinase, Aik, related to Aurora of Drosophila and yeast Ipl1. *J Biol Chem* **272**: 13766–13771

Nakayama H, Yokoi H and Fujita J (1992) Quantification of mRNA by non-radioactive RT-PCR and CCD imaging system. *Nucleic Acids Res* **25**: (20) 4939

Niwa H, Yamamura K and Miyazaki J (1991) Efficient selection for high-expression transfectants with a novel eukaryotic vector. *Gene* **15**: (108) 193–199

Ohta H, Sweeney EA, Masamune A, Yatomi Y, Hakomori S and Igarashi Y (1995) Induction of apoptosis by sphingosine in human leukemic HL-60 cells: a possible endogenous modulator of apoptotic DNA fragmentation occurring during phorbol ester-induced differentiation. *Cancer Res* **55**: 691–697

Pitti RM, Marsters SA, Lawrence DA, Roy M, Kischkei FC, Dowd P, Huang A, Donahue CJ, Sherwood SW, Baldwin DT, Godowski PJ, Wood WI, Gurney AL, Hillan KJ, Cohen RL, Goddard AD, Botstein D and Ashkenazi A (1998) Genomic amplification of a decoy receptor for Fas ligand in lung and colon cancer. *Nature* **396**: 699–703

Ranzani GN, Pellegata NS, Previdere C, Saragoni A, Vio A, Maltoni M and Amadori D (1990) Heterogeneous protooncogene amplification correlates with tumor progression and presence of metastases in gastric cancer patients. *Cancer Res* **50**: 7811–7814

Ried T, Knutzen R, Steinbeck R, Blegen H, Schrock E, Heselmeyer K, Du Manoir S and Auer G (1996) Comparative genomic hybridization reveals a specific pattern of chromosomal gains and losses during the genesis of colorectal tumors. *Genes Chromosomes Cancer* **15**: 234 –245

Sakakura C, Ymaguchi-Iwai Y, Satake M, Bae SC, Takahashi A, Ogawa E, Hagiwara A, Takahashi T, Murakami A, Makino K, Nakagawa AT, Kamada N and Ito Y (1994) Growth inhibition and induction of differentiation of t(8;21) acute myeloid leukemia cells by the DNA-binding domain of PEBP2 and the AML1/MTG8(ETO)-specific antisense oligonucleotide. *Proc Natl Acad Sci USA* **91**: 11723–11727

Sakakura C, Mori T, Sakabe T, Ariyama Y, Shinomiya T, Date K, Hagiwara A, Yamaguchi T, Takahashi T, Nakamura Y, Abe T and Inazawa J (1999) Gains, losses, and amplifications of genomic materials in primary gastric cancers analyzed by comparative genomic hybridization. *Genes Chromosomes Cancer* **24**: 299–305

Sakakura C, Sweeney EA, Shirahama T, Igarashi Y, Hakomori S, Nakatani H, Tsujimoto H, Imanishi T, OhgakiH M, Ohyama T, Yamazaki J and Hagiwara A, Yamaguchi T, Sawai K and Takahashi T (1996a) Overexpression of bax sensitizes human breast cancer MCF- 7 cells to radiation-induced apoptosis. *Int J Cancer* **67**: 101–105

Sakakura C, Sweeney EA, Shirahama T, Hakomoro S and Igarashi Y (1996b) Suppression of bcl-2 gene expression by sphingosine in the apoptosis of human leukemic HL-60 cells during phorbol ester-induced terminal differentiation. *FEBS Lett* **379**: 177–180

Sakakura C, Hagiwara A, Yasuoka R, Fujita Y, Nakanishi M, Maasuda K, Kimura A, Nakamura Y, Inazawa J, Abe T and Yamagishi H (2000) Amplification and overexpression of the AIB1 nuclear receptor co-activator gene in primary gastric cancers. *Int J Cancer* **89**: 217–223

Sen S, Zhou H and White RA (1997) A putative serine/threonine kinase encoding gene BTAK on chromosome 20q13 is amplified and overexpressed in human breast cancer cell lines. *Oncogene* **14**: 2195–2200

Shindo M, Nakano H, Kuroyanagi H, Shirasawa T, Mihara M, Gilbert DJ, Jenkins NA, Copeland NG, Yagita H and Okumura K (1998) cDNA cloning, expression, subcellular localization, and chromosomal assignment of mammalian aurora homologues, aurora-related kinase (ARK) 1 and 2.1. *Biochem Biophys Res Commun* **244**: 285–292

Stark GR, Debatisse M, Giulotto E and Wahl GM (1989) Recent progress in understanding mechanism of mammalian DNA amplification. *Cell* **57**: 901–908

Tahara E (1995) Molecular biology of gastric cancer. *World J Surg* **19**: 484–488

Tanner MM, Tirkkonen M, Kallioniemi A, Holli K, Collins C, Kowbel D, Gray JW, Kallioniemi O and Isola J (1995) Amplification of chromosomal region 20q13 in invasive breast cancer: prognostic implication. *Clin Cancer Res* **54**: 1455–1461

Tirkkonen M, Tanner M, Karhu R, Kallioniemi A, Isola J and Kallioniemi OP (1996) Molecular cytogenetics of primary breast cancer by CGH. *Genes Chromosomes Cancer* **21**: 177–184

Voorter C, Joos S, Bringuier PP, Vallinga M, Poddighe P, Schalken J and Du Manoir S, Ramaekers F, Lichter P and Hopman A (1995) Detection of chromosomal imbalances in transitional cell carcinoma of the bladder by comparative genomic hybridization. *Am J Pathol* **146**: 1341–1354

Wahl GM (1989) The importance of circular DNA in mammalian gene amplification. *Cancer Res* **49**: 1333–1340

Whelan SL, Parkin DM and Masuyer E (eds) (1993) Trends in Cancer Incidence and Mortality. IARC Scientific Publ No 102. IARC Lyon

Zhou H, Kuang J, Zhong L, Kuo WL, Gray JW, Sahin A, Brinkley BR and Sen S (1998) Tumour amplified kinase STK15/BTAK induces centrosome amplification, aneuploidy and transformation. *Nat Genet* **20**: 189–193

Zhuang Z, Park WS, Pack S, Schmidt L, Vortmeyer AO, Pak E, Pham T, Weil RJ, Candidus S, Lubensky IA, Linehan WM, Zbar B and Weirich G (1998) Trisomy 7-harbouring non-random duplication of the mutant MET allele in hereditary papillary renal carcinomas. *Nat Genet* **20**: 66 – 69

*© 2001 Cancer Research Campaign*
